# Combined classical cytogenetics and array Comparative Genomic Hybridisation for genomic copy number analysis

**DOI:** 10.1186/1755-8166-7-S1-P3

**Published:** 2014-01-21

**Authors:** Meena Lall, Pushpa Saviour, Ratna Puri, Preeti Paliwal, Surbhi Mahajan, Ishwar Verma

**Affiliations:** 1 Center of Medical Genetics, Sir Gangaram Hospital, New Delhi 110060, India

## Background

There is a great need for reporting and cataloging of genotype phenotype correlation of clusters of individuals sharing similar genomic rearrangements and phenotypes. This will facilitate diagnosis and personalized genetic counseling and will also improve our understanding of gene function and disease. Karyotyping and FISH are microscopic classical cytogenetic methods of detecting gain, loss or rearrangement of genetic material. Array Comparative Genomic Hybridisation (CGH) can used for molecular characterization, to size the abnormality and study the gene content.

## Materials and methods

A pilot study was designed: Combined classical cytogenetic, Array CGH and phenotypic findings were correlated in 20 patients with autism, mental retardation or congenital malformations.

## Results

Ten out of 20 patients with autism, mental retardation or congenital malformations had a normal karyotype and ten had abnormal karyotype. Ten normal subjects were included as normal controls, of which two had balanced translocations, which were not recognized by ACGH.

## Conclusions

Copy number variations detected by ACGH can be novel or extremely rare such that uncertainty may remain as to whether the aberration is pathogenic or simply a benign variant. Copy number variant (CNV) loci have been listed in the database of genomic variants (DGV). Using this and the UCSC browser the analysis of the size of the aberration and gene content was done in all individual cases, with normal or abnormal karyotypes. These findings were correlated with the phenotype to recognize individual syndromes.

## Conclusions

This pilot study is just the beginning. Identification of more patients, who share a region of genomic duplication or deletion and have phenotypic features in common, will allow greater certainty to be given to the pathogenic nature of the rearrangement and delineation of new syndromes. Most data available is western. Therefore there is a need to document and register Indian data accumulated to recognize the more common syndromes affecting our population and the common benign CNVs which can be discounted in our population, during the CNV analysis.

